# Feasibility of surface‐guidance combined with CBCT for intra‐fractional breath‐hold motion management during Ethos RT

**DOI:** 10.1002/acm2.14242

**Published:** 2024-01-04

**Authors:** Taeho Kim, Eric Laugeman, Kendall Kiser, Joshua Schiff, Shanti Marasini, Alex Price, H Michael Gach, Nels Knutson, Pamela Samson, Clifford Robinson, Casey Hatscher, Lauren Henke

**Affiliations:** ^1^ Radiation Oncology Washington University School of Medicine Washington USA; ^2^ Radiology and Biomedical Engineering Washington University School of Medicine Washington USA; ^3^ Radiation Oncology University Hospitals Case Western Reserve University

**Keywords:** adaptive radiotherapy, ethos, optical surface imaging, respiratory motion management

## Abstract

**Purpose:**

High‐quality CBCT and AI‐enhanced adaptive planning techniques allow CBCT‐guided stereotactic adaptive radiotherapy (CT‐STAR) to account for inter‐fractional anatomic changes. Studies of intra‐fractional respiratory motion management with a surface imaging solution for CT‐STAR have not been fully conducted. We investigated intra‐fractional motion management in breath‐hold Ethos‐based CT‐STAR and CT‐SBRT (stereotactic body non‐adaptive radiotherapy) using optical surface imaging combined with onboard CBCTs.

**Methods:**

Ten cancer patients with mobile lower lung or upper abdominal malignancies participated in an IRB‐approved clinical trial (Phase I) of optical surface image‐guided Ethos CT‐STAR/SBRT. In the clinical trial, a pre‐configured gating window (± 2 mm in AP direction) on optical surface imaging was used for manually triggering intra‐fractional CBCT acquisition and treatment beam irradiation during breath‐hold (seven patients for the end of exhalation and three patients for the end of inhalation). Two inter‐fractional CBCTs at the ends of exhalation and inhalation in each fraction were acquired to verify the primary direction and range of the tumor/imaging‐surrogate (donut‐shaped fiducial) motion. Intra‐fractional CBCTs were used to quantify the residual motion of the tumor/imaging‐surrogate within the pre‐configured breath‐hold window in the AP direction. Fifty fractions of Ethos RT were delivered under surface image‐guidance: Thirty‐two fractions with CT‐STAR (adaptive RT) and 18 fractions with CT‐SBRT (non‐adaptive RT). The residual motion of the tumor was quantified by determining variations in the tumor centroid position. The dosimetric impact on target coverage was calculated based on the residual motion.

**Results:**

We used 46 fractions for the analysis of intra‐fractional residual motion and 43 fractions for the inter‐fractional motion analysis due to study constraints. Using the image registration method, 43 pairs of inter‐fractional CBCTs and 100 intra‐fractional CBCTs attached to dose maps were analyzed. In the motion range study (image registration) from the inter‐fractional CBCTs, the primary motion (mean ± std) was 16.6 ± 9.2 mm in the SI direction (magnitude: 26.4 ± 11.3 mm) for the tumors and 15.5 ± 7.3 mm in the AP direction (magnitude: 20.4 ± 7.0 mm) for the imaging‐surrogate, respectively. The residual motion of the tumor (image registration) from intra‐fractional breath‐hold CBCTs was 2.2 ± 2.0 mm for SI, 1.4 ± 1.4 mm for RL, and 1.3 ± 1.3 mm for AP directions (magnitude: 3.5 ± 2.1 mm). The ratio of the actual dose coverage to 99%, 90%, and 50% of the target volume decreased by 0.95 ± 0.11, 0.96 ± 0.10, 0.99 ± 0.05, respectively. The mean percentage of the target volume covered by the prescribed dose decreased by 2.8 ± 4.4%.

**Conclusion:**

We demonstrated the intra‐fractional motion‐managed treatment strategy in breath‐hold Ethos CT‐STAR/SBRT using optical surface imaging and CBCT. While the controlled residual tumor motion measured at 3.5 mm exceeded the predetermined setup value of 2 mm, it is important to note that this motion still fell within the clinically acceptable range defined by the PTV margin of 5 mm. Nonetheless, additional caution is needed with intra‐fractional motion management in breath‐hold Ethos CT‐STAR/SBRT using optical surface imaging and CBCT.

## 1 INTRODUCTION

High‐quality cone beam computed tomography (CBCT) and artificial intelligence (AI)‐enhanced adaptive planning techniques on the Ethos System (Varian Medical Systems, CA) allow for inter‐fractional motion management via CBCT‐guided stereotactic adaptive radiotherapy (CT‐STAR).^1^, ^2^, ^3^, ^4^ The inter‐fractional management has been demonstrated in silico and/or clinically in a variety of disease sites.^2^, ^5^, ^6^, ^7^ In contrast to inter‐fractional changes, intra‐fractional changes require continual management during each fraction of the treatment course.^8^ In intra‐fractional management, respiratory motion control is a dominant element in managing motion‐related image artifacts and tumor motion.^9^, ^10^, ^11^

Surface imaging, in general, has been actively used for routine patient positioning and is effective for deep‐inspiration breath‐hold (DIBH) treatment in radiation therapy (surface‐guided radiation therapy: SGRT).^12^, ^13^, ^14^, ^15^ For example, Naumann et al. reported a surface‐guided position verification and monitoring combined with CBCT for stereotactic body radiotherapy (SBRT)‐DIBH of 10 patients (3 lung and 7 liver cases).^16^ In addition, surface imaging was used to manage intra‐fractional respiratory‐related motion.^14^, ^17^ Li et al. reported that surface imaging could characterize respiratory motion and provide a good respiratory surrogate.^18^ Nonionizing surface imaging with high spatial and temporal resolution is beneficial for continuous monitoring with real‐time feedback during treatment as well as inter‐fractional patient positioning.^19^ Surface imaging systems utilize the temporal surfaces of a patient compared to a reference surface using optical imaging technology. Although surface imaging of a patient is acquired at the treatment isocenter, target verification using radiographic 2D or 3D imaging is a common practice, especially for deeply positioned targets.^12^

CT‐STAR was demonstrated to account for inter‐fractional anatomic changes.^2^, ^5^, ^20^, ^21^ However, intra‐fractional respiratory motion management on the Ethos system has been a practical challenge due to the closed‐bore ring gantry LINAC and offset kV panel of the Ethos system.^22^ To the authors’ knowledge, there is limited literature available on the topic of intra‐fractional respiratory motion management with a surface imaging solution for Ethos stereotactic body adaptive radiotherapy.^23^, ^24^ Recently, our institution incorporated the SGRT system, IDENTIFY (Varian Medical Systems, CA), with the Ethos system. Thus, we investigated intra‐fractional motion‐management strategies in breath‐hold for Ethos treatments using optical surface imaging in conjunction with on‐board CBCTs in a Phase I clinical trial (NCT05030454).

In this report, we present our institutional study results for intra‐fractional motion management to aid breath‐hold control in CBCT scans and beam delivery in Ethos CT‐STAR (stereotactic adaptive radiotherapy) and CT‐SBRT (stereotactic non‐adaptive radiotherapy). The study measured the: (1) inter‐fractional tumor motion range, (2) intra‐fractional tumor motion in breath‐hold, and (3) dosimetric impact on dose coverage for tumor by surface‐guided intra‐fractional motion management.

## 2 METHODS

### 2.1 Pilot study of a surface image‐guided Ethos CT‐STAR/SBRT

Ten cancer patients with mobile lower lung or upper abdominal malignancies participated in an IRB‐approved clinical trial (Phase I) of optical surface image‐guided Ethos CT‐STAR/SBRT (Table 1). We included patients who have primary or metastatic disease of the abdomen or lower thorax, with biopsy‐proven or radiographically diagnosed disease histology of solid tumor categorization, with the exception of small cell cancers. The study did not include the patients who have primary disease of hematologic origin, lymphoma, or small cell cancer. Three male and seven female patients were in the clinical trial (age mean ± std: 70.4 ± 13.4 years). According to medical considerations such as breath‐hold capability, tumor location, and tumor mobile range, seven patients were treated with breath‐hold at the end of exhalation and three patients with breath‐hold at the end of inhalation (total 50 fractions: 32 CT‐STAR and 18 CT‐SBRT). The expiration breath‐hold was preferred due to the position reproducibility and less compression to abdominal tumors. However, the inspiration breath‐hold approach was used if the patient could not tolerate the exhalation breath‐hold for their treatment. Also, the inspiration breath‐hold treatment was conducted for a lung case because of its superior lung tissue sparing. The treatment locations included seven pancreases, one liver, one retroperitoneal space, and one lung. Three patients received non‐adaptive SBRT based on the distance between the critical structures and targets. Additionally, we switched one patient from the adaptive treatment to the non‐adaptive treatment due to patient intolerance to a long adaptive procedure.

**TABLE 1:** Patient, disease, and treatment characteristics.

	Sex	Age	Disease site	Breath‐hold	Rx (Gy)	# of Fractions	# of CT‐STAR Fractions
**P1**	M	76	Pancreas	Exhale	50	5	2
**P2**	F	40	Retroperitoneal space	Inhale	50	5	0
**P3**	F	84	Pancreas	Exhale	50	5	5
**P4**	F	73	RT Lung	Inhale	55	5	0
**P5**	F	73	Pancreas	Exhale	50	5	5
**P6**	F	87	Pancreas	Exhale	50	5	5
**P7**	M	75	Pancreas	Exhale	50	5	5
**P8**	M	72	Pancreas	Exhale	50	5	5
**P9**	F	59	Liver	Inhale	50	5	0
**P10**	F	65	Pancreas	Exhale	50	5	5

*Note*: P: Patient, M: Male, F: Female, Exhale: Breath‐hold at the end of exhalation, Inhale: Breath‐hold at the end of inhalation, Rx: Prescription radiation dose in Gy, # of Fraction: Total number of treatment fractions, and # of CT‐STAR Fractions: Total number of CT‐STAR fractions.

### 2.2 Workflow of surface image‐guided Ethos CT‐STAR/SBRT

Each patient received patient‐specific respiratory motion management during the course of treatment. In each fraction, Ethos CT‐STAR/SBRT workflow consisted of two initial CBCTs in breath‐hold (at the end of inhalation and exhalation), and a breath‐hold CBCT before each arc delivery that matched the breath‐hold position of the primary image dataset (one of two initial CBCTs). In instances where patients exceeded the predefined gating window (±2 mm in the AP direction) for an extended period or experienced significant positional shifts, we temporarily halted the treatment and performed a repeat breath‐hold CBCT to confirm their position. Consequently, the number of intra‐fractional CBCT scans might vary.

**FIGURE 1:**
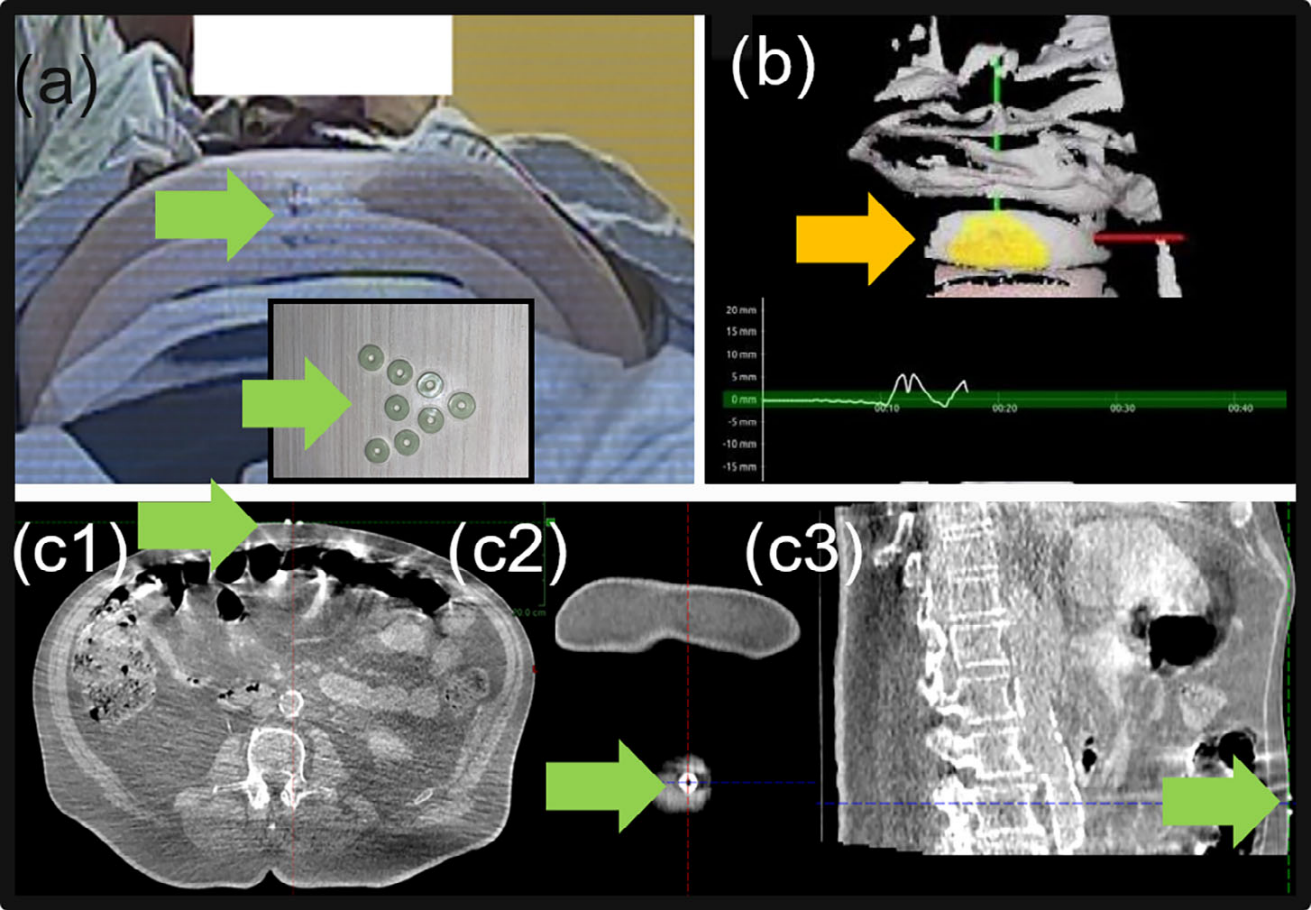
Components of the surface image‐guided Ethos CT‐STAR/SBRT. (a) donut‐shaped imaging‐surrogate on patient's abdomen (green arrow), (b) auxiliary software with real‐time monitoring display (orange arrow for ROI on the abdomen), and (c) donut‐shaped imaging‐surrogate on CBCT. c1: axial, c2: coronal, and c3: sagittal views.

IDENTIFY was employed as a surface imaging system for respiratory motion management. Compared to the system for open rotating gantry LINAC,^12^ the modified IDENTIFY system had two camera pods in front of the bore and one camera pod on the back wall. Each camera pod included structured light projection with two cameras, achieving sub‐mm accuracy. For IDENTIFY with Ethos radiotherapy system, the radiation isocenter calibration was not supported. Therefore, a new reference surface was acquired at the beginning of each treatment session and only used for intra‐fraction motion monitoring. Based on the measurements using a cubic phantom moving in all directions, the vertical, lateral, and longitudinal ranges of the IDENTIFY are 31.73 cm, 39.85 cm, and 112.19 cm. In the clinical workflow, auxiliary software was used to display the temporal and reference surfaces of a patient. The display was color‐coded to aid the clinical team during patient setup. In addition, a real‐time feedback interface was used to monitor the region of interest (ROI) continuously (Figure 1b: yellow), but it did not interlock the beam system.

A pre‐configured gating window (±2 mm in AP direction) on the optical surface imaging was used for manually triggering the intra‐fractional CBCT and treatment beam irradiation during breath‐hold (Figure 1b: orange arrow). A donut‐shaped imaging surrogate placed on the patient's abdomen, visible in the CBCT, served to verify the patient's abdominal positioning, as depicted in Figure 1a (green arrow). As the surface imaging system operated independently of the CBCT system, we incorporated the imaging surrogate to verify the abdominal position in relation to the tumor's location within the clinical Ethos CT‐STAR/SBRT workflow (Figure 1c with the green arrow).

### 2.3 Inter‐ and intra‐fractional tumor motions

For the tumor motion analysis, breath‐held CBCTs were assessed with surface‐guided respiratory motion management in place. In each fraction, two initial CBCTs in breath‐hold at the end of inhalation and the end of exhalation were assessed to determine the tumor/imaging‐surrogate motion ranges. Intra‐fractional breath‐held CBCTs were used to quantify the position difference of the tumor/imaging‐surrogate (residual motion) within the pre‐configured gating window of IDENTIFY from the reference position (primary image dataset).

**FIGURE 2:**
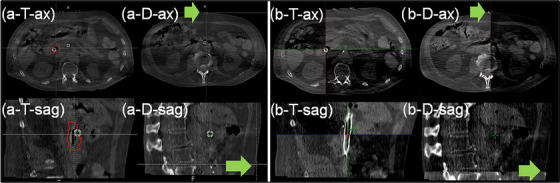
Tumor and donut‐shaped imaging‐surrogate identification on CBCTs. (a) positions of tumor centroids and donut‐shaped imaging‐surrogates were manually identified by Radiation Oncologists using the Ethos TPS, and (b) each CBCT (bright contrast) was registered to the fractional reference CBCT (darker contrast) by tumor and donut‐shaped imaging‐surrogate in ARIA. T: tumor, D: donut‐shaped imaging‐surrogate, ax: axial, sag: sagittal views. Red contour indicates the GTV. The green arrow points to the donut‐shaped imaging‐surrogate.

**Method 1‐ manual mapping**: Positions of tumor centroid and donut‐shaped imaging‐surrogates were manually identified by Radiation Oncologists in the Ethos treatment planning system (TPS) shown in Figure 2a. The coordinates for each ROI (tumor centroid and donut‐shaped imaging‐surrogate) were recorded, and the coordinate difference between CBCTs and the reference CBCT in x‐, y‐, and z‐direction were calculated and reported. The x‐axis corresponds to the Right‐Left (RL) direction, the y‐axis corresponds to the Anterior‐Posterior (AP) direction, and the z‐axis corresponds to the Superior‐Inferior (SI) direction.

**Method 2‐ image registration**: Each Ethos session was exported and all data (CBCTs, structure sets, doses) were imported to ARIA (Oncology Information System, Varian Medical Systems, CA, USA). All CBCTs taken on Ethos system have the imaging isocenter set as the DICOM origin of the image. Therefore, CBCTs were co‐registered based on DICOM origin, which is fixed in the LINAC frame of reference. In the Image Registration workspace, each CBCT was registered to the fractional reference CBCT by tumors and donut‐shaped imaging‐surrogates individually shown in Figure 2b. The image translations of each CBCT from the reference CBCT in x‐, y‐, and z‐direction were calculated and reported. No rotational registration was applied due to tumor shape complexity caused by intrinsic shape, rotational changes, and deformation of the tumor during CBCT.

Quantitative statistical comparisons of the intra‐fractional residual motion magnitudes between the tumor and donut across all fractions, including fractions 1 to 5 were conducted using the unpaired Student's t‐test (Microsoft Excel 2016, TTEST).

### 2.4 Dosimetric impact on tumor coverage by surface‐guided intra‐fractional motion management

Within the Ethos TPS, a partial dose map was computed for each CBCT scan following arc delivery. Subsequently, the cumulative dose map was generated by aligning each CBCT with the reference CBCT and consolidating the partial dose maps onto the reference CBCT. We shifted each partial dose map from the treatment isocenter by conducting a soft‐tissue rigid registration of CBCTs with the registration window focused on the tumor in Aria. Therefore, the combined dose map deviated from the planning dose. Dosimetric deviation of the dose coverage for tumor was assessed.

## 3 RESULTS

Fifty fractions of Ethos RT were delivered under surface imaging‐guidance: 32 fractions with CBCT‐guided CT‐STAR and 18 fractions with CT‐SBRT (total 50 fractions). As a result of technical challenges, access to four of the treatment records in the Ethos TPS was unavailable after treatment, leading to the utilization of 46 fractions for the analysis of intra‐fractional residual motion. Furthermore, in three fractions of 46, two initial breath‐hold CBCTs (one at the end of inhalation and another at exhalation) were not acquired. This resulted in the use of 43 fractions for inter‐fractional motion analysis.

### 3.1 Inter‐ and intra‐fractional tumor motions using manual mapping

The following are the results of the tumor motion using the manual mapping method.

#### 3.1.1 Inter‐fractional motion of tumor and donut‐shaped imaging‐surrogate

Three pairs of 46 datasets were not processed due to system issues. A total of 43 pairs of CBCTs were assessed. The ranges of the tumor motion and the donut‐shaped imaging‐surrogate motion are presented in Table 2. The values of mean ± std were 16.8 ± 11.3 mm in the SI direction for the tumors and 16.3 ± 7.3 mm in the AP direction for the donut‐shaped imaging‐surrogate, respectively (large motion in gray highlight).

**TABLE 2:** Inter‐fractional motion (manual mapping): Summary (mean ± std) of inter‐fractional motion range for tumor and donut‐shaped imaging‐surrogate (donut).

	SI (mm)	RL (mm)	AP (mm)	Mag (mm)
**Tumor**	16.8 ± 11.3	7.5 ± 5.0	12.5 ± 9.8	25.7 ± 12.1
**Donut**	7.9 ± 6.7	2.4 ± 2.1	16.3 ± 7.3	20.0 ± 7.1
***p*‐value**				0.04

*Note*: RL: Right‐Left direction, AP: Anterior‐Posterior direction, SI: Superior‐Inferior (large motion in gray highlight), and Mag: Magnitude.

#### 3.1.2 Intra‐fractional residual motion of tumor and donut‐shaped imaging‐surrogate

A total of 120 CBCTs were assessed. Intra‐fractional residual motion of tumor and donut‐shaped imaging‐surrogate based on fraction are presented in Table 3. The residual motion of the tumors was 1.8 ± 1.8 mm for SI, 2.0 ± 2.2 mm for RL, and 2.0 ± 1.8 mm for AP directions. The residual motion of the donut‐shaped imaging‐surrogate from intra‐fractional CBCTs was 2.3 ± 2.1 mm for SI, 2.5 ± 2.0 mm for RL, and 2.0 ± 2.3 mm for AP directions.

**TABLE 3:** Intra‐fractional motion (manual mapping) based on fraction, CBCT, and breath‐hold mode: Mean ± std, RL: Right‐Left direction, AP: Anterior‐Posterior direction, SI: Superior‐Inferior, and Mag: Magnitude.

	Tumor (mm)				Donut (mm)			
FX (# CBCT)	SI	RL	AP	Mag	SI	RL	AP	Mag (p‐value)
**All (120)**	1.8 ± 1.8	2.0 ± 2.2	2.0 ± 1.8	4.0 ± 2.5	2.3 ± 2.1	2.5 ± 2.0	2.0 ± 2.3	4.6 ± 2.8 (0.57)
**Fx1 (26)**	1.6 ± 1.4	1.9 ± 1.5	3.1 ± 2.8	4.6 ± 2.7	2.2 ± 1.8	2.1 ± 1.7	1.8 ± 1.7	4.0 ± 2.2 (0.25)
**Fx2 (22)**	2.1 ± 1.6	3.3 ± 3.8	1.7 ± 1.6	4.9 ± 3.7	3.3 ± 2.2	2.7 ± 2.4	1.7 ± 2.1	5.2 ± 2.9 (0.71)
**Fx3 (22)**	2.3 ± 1.8	1.7 ± 1.7	1.8 ± 1.4	4.1 ± 1.7	1.6 ± 2.4	1.6 ± 1.6	1.7 ± 1.9	3.3 ± 2.9 (0.31)
**Fx4 (24)**	1.8 ± 2.0	1.6 ± 1.7	1.6 ± 1.3	3.5 ± 2.1	2.2 ± 1.9	2.0 ± 1.8	2.1 ± 2.8	4.4 ± 3.0 (0.32)
**Fx5 (26)**	1.1 ± 1.8	1.8 ± 1.5	1.6 ± 1.1	3.1 ± 1.9	1.7 ± 1.6	2.7 ± 2.2	2.2 ± 2.4	4.5 ± 2.7 (0.04)
**# CBCT**								
**CBCT‐1 (46)**	1.7 ± 1.5	1.8 ± 2.3	1.8 ± 1.7	3.7 ± 2.5	1.8 ± 1.7	2.2 ± 2.1	1.9 ± 2.2	4.1 ± 2.7
**CBCT‐2 (43)**	2.0 ± 2.1	1.9 ± 1.8	1.6 ± 1.8	3.8 ± 2.5	2.0 ± 1.7	2.1 ± 1.7	1.7 ± 2.3	4.0 ± 2.6
**CBCT‐3 (20)**	1.6 ± 1.6	2.8 ± 2.8	2.3 ± 1.6	4.5 ± 2.9	2.7 ± 2.2	2.6 ± 1.9	2.1 ± 1.9	4.9 ± 2.6
**CBCT‐4 (6)**	1.8 ± 1.3	1.6 ± 2.3	2.7 ± 1.1	4.3 ± 1.5	2.3 ± 2.9	2.8 ± 3.4	2.7 ± 2.7	5.1 ± 4.5
**CBCT‐5 (3)**	1.3 ± 1.2	1.8 ± 0.9	4.1 ± 3.2	4.8 ± 3.1	6.0 ± 2.0	2.1 ± 2.0	2.0 ± 2.7	7.2 ± 2.3
**CBCT‐6 (2)**	2.0 ± 2.8	3.4 ± 2.0	5.3 ± 3.4	7.2 ± 2.6	3.0 ± 4.2	1.0 ± 1.3	1.0 ± 1.3	4.1 ± 3.1
**Breath‐hold (# of patients)**								
**Exhale (7)**	1.8 ± 1.8	2.2 ± 2.4	2.0 ± 1.9	4.2 ± 2.6	2.0 ± 2.1	2.3 ± 2.1	2.0 ± 2.4	4.4 ± 2.9
**Inhale (3)**	1.6 ± 1.7	1.5 ± 1.5	1.9 ± 1.8	3.4 ± 2.2	2.6 ± 1.9	2.0 ± 1.8	1.5 ± 1.7	4.1 ± 2.4

*Note*: Fx: Fraction (Intra‐fractional motion from all CBCTs in gray highlight).

Intra‐fractional residual motion of the tumor and donut‐shaped imaging‐surrogate based on CBCT and breath‐hold mode are presented in Table 3. The number of intra‐fractional CBCT scans varied as a result of variations in patient compliance with both breath‐hold requirements and treatment. The number of the first CBCT (CBCT‐1) and the second CBCT (CBCT‐2) are 45 and 43 sets. In contrast, the number of the fifth CBCT (CBCT‐5) and the sixth (CBCT‐6) are 3 and 2 sets. The residual motion of the tumor from CBCT‐5 and CBCT‐6 show similar variation compared to those from CBCT‐1 and CBCT‐2. However, the residual motion of the donut‐shaped imaging‐surrogate from CBCT‐5 and CBCT‐6 showed large variations compared to those from CBCT‐1 and CBCT‐2. In a comparison of the intra‐fractional residual motion magnitudes between the tumor and donut across all fractions, including fractions 1 to 5, the mean residual motion of the tumor and the donut‐shaped imaging‐surrogate were less than 5 mm in magnitude (*p* > 0.05).

### 3.2 Inter‐ and intra‐fractional tumor motions using image registration

The results of the tumor motion using the image registration method are presented.

#### 3.2.1 Inter‐fractional motion of tumor and donut‐shaped imaging‐surrogate

A total of 43 pairs of CBCTs were assessed. The ranges of the tumor motion and the donut‐shaped imaging‐surrogate motion are presented in Table 4. The values of mean ± std were 16.6 ± 9.2 mm in the SI direction for the tumors and 15.5 ± 7.3 mm in the AP direction for the donut‐shaped imaging‐surrogate, respectively (large motion in gray highlight).

**TABLE 4:** Inter‐fractional motion (image registration): Summary (mean ± std) of inter‐fractional motion range for tumor and donut‐shaped imaging‐surrogate (donut).

	SI (mm)	RL (mm)	AP (mm)	Mag (mm)
**Tumor**	16.6 ± 9.2	7.4 ± 6.9	12.4 ± 10.4	26.4 ± 11.3
**Donut**	9.0 ± 6.0	3.4 ± 2.5	15.5 ± 7.3	20.4 ± 7.0
***p*‐value**				0.01

*Note*: RL: Right‐Left direction, AP: Anterior‐Posterior direction, SI: Superior‐Inferior (large motion in gray highlight), and Mag: Magnitude.

#### 3.2.2 Intra‐fractional residual motion of tumor and donut‐shaped imaging‐surrogate

A total of 100 CBCTs attached to the dose maps were assessed. Intra‐fractional residual motion of tumor and donut‐shaped imaging‐surrogate based on fraction are presented in Table 5. The residual motion of the tumors was 2.2 ± 2.0 mm for SI, 1.4 ± 1.4 mm for RL, and 1.3 ± 1.3 mm for AP directions. The residual motion of the donut‐shaped imaging‐surrogate from intra‐fractional CBCTs was 2.2 ± 2.3 mm for SI, 1.8 ± 1.7 mm for RL, and 1.9 ± 2.2 mm for AP directions.

**TABLE 5:** Intra‐fractional motion (image registration) based on fraction, CBCT, and breath‐hold mode: Mean ± std, RL: Right‐Left direction, AP: Anterior‐Posterior direction, SI: Superior‐Inferior, and Mag: Magnitude.

	Tumor (mm)				Donut (mm)			
FX (# CBCT)	SI	RL	AP	Mag	SI	RL	AP	Mag (p‐value)
**All (100)**	2.2 ± 2.0	1.4 ± 1.4	1.3 ± 1.3	3.5 ± 2.1	2.2 ± 2.3	1.8 ± 1.7	1.9 ± 2.2	4.2 ± 2.7 (0.06)
**Fx1 (21)**	2.8 ± 2.3	1.6 ± 1.7	1.6 ± 1.7	4.3 ± 2.4	1.6 ± 1.9	2.1 ± 2.1	1.7 ± 1.4	3.9 ± 2.0 (0.27)
**Fx2 (18)**	2.6 ± 2.7	1.8 ± 1.4	1.1 ± 1.0	3.8 ± 2.6	2.6 ± 2.1	1.9 ± 1.9	1.9 ± 2.1	4.5 ± 2.6 (0.43)
**Fx3 (20)**	2.3 ± 2.0	1.4 ± 0.8	1.7 ± 1.2	3.6 ± 1.7	2.6 ± 2.8	1.4 ± 1.5	1.1 ± 1.1	3.6 ± 2.8 (0.97)
**Fx4 (16)**	2.0 ± 1.4	1.6 ± 2.3	1.3 ± 1.1	3.3 ± 2.4	1.9 ± 2.2	1.5 ± 1.5	2.0 ± 3.9	4.0 ± 4.1 (0.54)
**Fx5 (25)**	1.7 ± 1.1	0.9 ± 0.7	1.0 ± 1.1	2.6 ± 1.1	2.4 ± 2.2	2.1 ± 1.7	2.5 ± 1.9	4.7 ± 2.3 (< 0.01)
**# CBCT**								
**CBCT‐1 (46)**	2.0 ± 1.8	1.3 ± 1.5	1.2 ± 1.0	3.2 ± 1.8	2.1 ± 1.9	1.8 ± 1.6	1.5 ± 1.5	3.7 ± 2.1
**CBCT‐2 (40)**	2.7 ± 2.2	1.4 ± 1.5	1.4 ± 1.5	3.8 ± 2.5	2.0 ± 2.0	1.8 ± 1.7	2.1 ± 2.9	4.2 ± 3.1
**CBCT‐3 (11)**	1.4 ± 1.3	1.8 ± 1.4	1.1 ± 1.3	3.0 ± 1.6	2.5 ± 2.3	2.4 ± 2.5	2.5 ± 2.0	5.2 ± 2.4
**CBCT‐4 (3)**	1.9 ± 2.9	2.0 ± 0.7	2.9 ± 1.3	4.7 ± 1.5	6.2 ± 6.1	0.9 ± 0.5	2.4 ± 0.3	7.3 ± 5.0
**Breath‐hold (# of patients)**								
**Exhale (7)**	2.4 ± 1.9	1.3 ± 1.2	1.3 ± 1.3	3.4 ± 2.1	2.3 ± 2.4	1.8 ± 1.7	2.0 ± 2.4	4.4 ± 2.9
**Inhale (3)**	1.9 ± 2.0	1.7 ± 1.9	1.5 ± 1.3	3.7 ± 2.2	1.8 ± 1.6	1.5 ± 1.8	1.3 ± 1.4	3.3 ± 2.0

*Note*: Fx: Fraction (Intra‐fractional motion from all CBCTs in gray highlight).

Intra‐fractional residual motion of tumor and donut‐shaped imaging‐surrogate based on CBCT and breath‐hold mode are presented in Table 5. The number of the first CBCT (CBCT‐1) and the second CBCT (CBCT‐2) are 46 and 40 sets. In contrast, the number of the third CBCT (CBCT‐3) and the fourth (CBCT‐4) are 11 and 3 sets. The residual motion of the tumor from CBCT‐3 and CBCT‐4 show similar variation compared to those from CBCT‐1 and CBCT‐2 compared to a large variation for the donut‐shaped imaging‐surrogate. In a comparison of the intra‐fractional residual motion magnitudes between the tumor and donut across all fractions, including fractions 1 to 5, the mean residual motion of the tumor and the donut‐shaped imaging‐surrogate were less than 5 mm in magnitude (*p* > 0.05).

**FIGURE 3:**
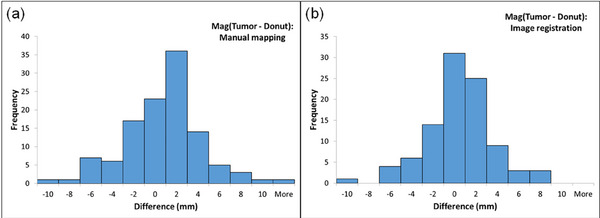
Histogram of the differences between the magnitudes of tumor shifts and donut‐shaped imaging‐surrogate shifts using (a) manual mapping and (b) image registration for all intra‐fractional CBCTs. The differences present the correlations of the surface motions with the tumor motion within the gating window.

Figure 3 displays histograms illustrating the differences in magnitudes between tumor shifts and donut‐shaped imaging‐surrogate shifts utilizing both (a) manual mapping and (b) image registration methods across all intra‐fractional CBCTs. These histograms serve as a visual representation of the effectiveness of the surface as a surrogate for 3D tumor motion within the triggering in the AP direction. A summary of the differences (mean ± std) are −0.19 ± 3.65 mm for manual mapping and −0.63 ± 3.29 mm for image registration. This data indicates the absence of any systematic variations between the two methods. Such a result strengthens the confidence in the assertion that controlling 2 mm of surface AP motion corresponds to less than 5 mm of tumor motion.

### 3.3 Dosimetric impact on tumor coverage by surface‐guided intra‐fractional motion management

**FIGURE 4:**
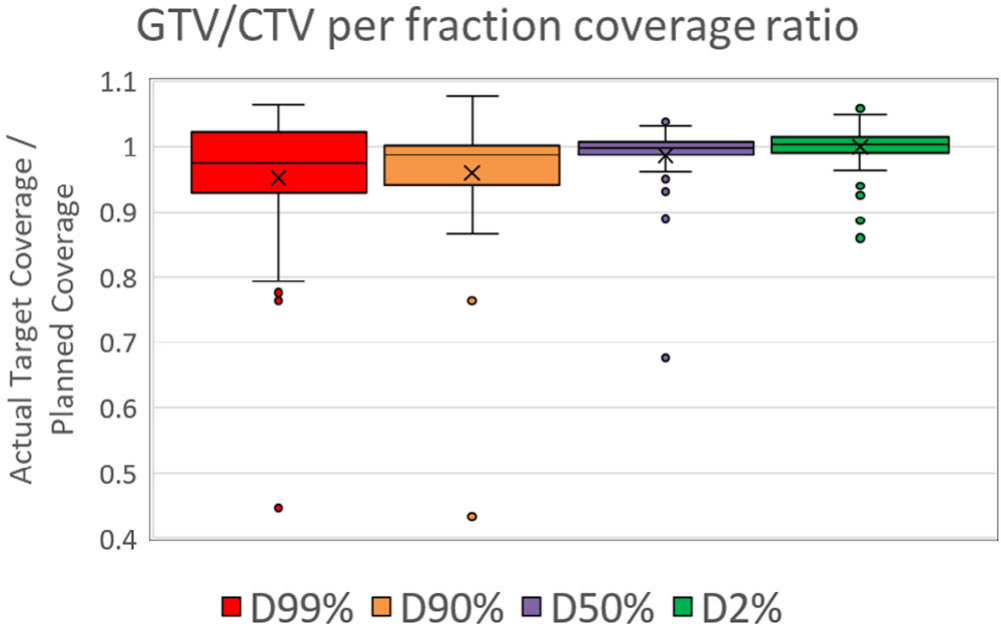
Ratio of actual target coverage from combined dose map using CBCT before each arc to planned target coverage for various metrics. DX%: Dose to X% of target volume.

Figure 4 shows a plot of the ratio of the actual target coverage from the combined dose map compared to the planned dose. The ratio of the actual dose coverage to 99%, 90%, and 50% of the target volume decreased by 0.95 ± 0.11, 0.96 ± 0.10, 0.99 ± 0.05. The mean ratio of dose to 2% of the target volume was 1.00 ± 0.04. The mean percentage of the target volume covered by prescribed dose decreased by 2.8% ± 4.4%.

## 4 DISCUSSION

This study presented the clinical trial results of surface image‐guided Ethos CT‐STAR/SBRT with ten cancer patients at a single institution. We investigated intra‐fractional residual tumor motion using on‐board CBCTs combined with surface image‐guided breath‐hold. We found the residual motion of the tumors was comparable to the pre‐determined gating window. The proposed intra‐fractional motion‐management strategy can be easily applied and personalized for Ethos CT‐STAR/SBRT, and extended to another disease site.

The benefits of the surface imaging include its simplicity and applicability during Ethos CT‐STAR/SBRT. As shown in Figure 1, the auxiliary software interactively guided the clinical team during patient setup and motion monitoring. The continuous motion monitoring was conducted with two camera pods in front of the bore and one camera pod on the back wall without camera occlusion from the bore. In the ROI selection, we monitored a part of the abdominal area that presented active breathing motion in Figure 1. We found that monitoring the entire abdomen could diminish the correlation in amplitude between the respiratory surrogate and the tumor motion. The rationale behind this is that when encompassing the entire abdomen (and thorax), including both active and inactive regions aligned with respiratory tumor motion, the respiratory signal correlation can be weak compared to the actual tumor motion range. Therefore, rather than monitoring a large surface area to estimate tumor motion, it is more effective to focus on the active area directly correlated with tumor motion. An ROI in the active breathing area was preferred in the clinical trial since we used a tight gating window of 2 mm.

According to TG‐302, beam‐hold threshold selection of SGRT should be specific to the disease site or patient.^12^ For example, breast deep‐inspirational breath‐hold (DIBH) treatment is feasible with a 2−3° rotational and 3−5 mm translational beam‐hold threshold in each direction. Since our clinical trial employed respiratory management with the exhalation breath‐hold (7 patients) and the normal inhalation breath‐hold (3 patients), half of the breast DIBH beam‐hold threshold (2 mm in the vertical position) should be equally achievable. From the assessment of 100 intra‐fractional CBCTs attached to the dose maps, we found that the averaged intra‐fractional residual motion was 2.2 mm (SI), 1.4 mm (RL), and 1.3 mm (AP) for the tumors in surface image‐guided breath‐hold management (magnitude: 3.5 ± 2.1 mm). Our findings are less than those reported by Stanley et al., of 5 ± 3 mm and 6 ± 2 mm of CBCT‐3D shifts after surface imaging for the abdominal site and breast, respectively.^25^ The quantitative statistical comparisons of the intra‐fractional residual motion magnitude between the tumor and donut‐shaped imaging‐surrogate across all fractions, including fractions 1 to 5 were reported in Table 3 and 5. The mean residual motion magnitude of the tumor and the donut‐shaped imaging‐surrogate across all fractions were less than 5 mm, which is statistically insignificant (*p* > 0.05).

We identified two patients who required repeat intra‐fractional breath‐hold CBCT scans. When patients exceeded the predefined gating window for an extended period or encountered significant positional shifts, we conducted additional breath‐hold CBCT scans to verify their positioning. In Table 3, we observed two patients who underwent 6 intra‐fractional CBCT scans. One patient exhibited an intra‐fractional magnitude of 4.2 ± 1.5 mm (mean ± standard deviation), while the other patient displayed 6.5 ± 1.5 mm. These measurements exceeded the reference value of 4.0 ± 2.5 mm, which was calculated based on data from all 10 patients. In Table 5, we once again identified the same two patients who underwent 4 intra‐fractional CBCT scans. One of these patients demonstrated an intra‐fractional magnitude of 4.2 ± 0.9 mm (mean ± standard deviation), while the other patient exhibited 5.5 ± 1.6 mm. These measurements exceeded the reference value of 3.5 ± 2.1 mm in Table 5.

The dosimetry analysis showed a decrease in target coverage due to the residual motion (magnitude: 3.5 ± 2.1 mm). Each plan utilized a 5 mm isotropic PTV margin from the GTV or CTV. For CT‐STAR planning, PTV coverage can be sacrificed in order to avoid nearby Organs at Risk (OARs).^5^ Therefore, sharp dose gradients often are closer, or within the target, than typical SBRT planning. Despite this, the residual target motion using breath‐hold treatments resulted in acceptable dose coverage. The most extreme outliers seen in Figure 3 for D99% and D90% metrics were due to a SI misalignment of the target. This CT‐STAR patient was intended for adaptive RT but was switched to SBRT due to their inability to lay on the couch for the increased time required for adaptive treatments. This clinical experience reinforces the importance of screening patients for good candidates when using manually gated breath‐hold treatments.

The surface imaging has the benefits of being nonionizing with high spatial and temporal resolution and is well suited for continuous monitoring with real‐time feedback during treatment.^12^, ^19^ Although routine QA tests with rigid phantoms were performed to keep the accuracy and integrity of the system in compliance with TG‐302, the deformable surface of a patient can degrade the accuracy of the system during Ethos CT‐STAR/SBRT. Since we treated the thoracicabdominal area, the surface of the patient showed substantial deformation during breathing. We manually triggered the CBCT and treatment beam irradiation based on a pre‐configured gating window on the axillary software so we introduced the donut‐shaped imaging‐surrogate into the workflow to verify the surface position of a patient. As reported, we found that the averaged intra‐fractional residual motions were 2.2 mm (SI), 1.8 mm (RL), and 1.9 mm (AP) from 100 intra‐fractional CBCTs for the donut‐shaped imaging‐surrogate in good agreement with the beam‐hold threshold.

The proposed strategy has a few limitations. First, our finding only pertains to intra‐fractional residual tumor motion during breath‐hold. Therefore, the correlation between the surface and tumor respiratory motion in free breathing was not investigated or discussed in this report. Second, there was intrinsic uncertainty in the CBCT acquisition due to manual gating. Although the clinical team followed the procedure, operational mistakes and inter‐operator conditions can introduce uncertainty to the results. Third, tumor dose coverage assumed rigid translation of the target. Any deformations of the tumor are not accounted for, nor represented in the dose coverage analysis. Fourth, we only investigated the tumor dose coverage, not OAR sparing. Since organ motion can be affected by many factors such as respiration, peristalsis, and organ filling (bladder, stomach, rectum), we do not believe that the proposed strategy can manage all organ motion. Further investigations are needed regarding organ motion management. Fifth, the IDENTIFY system and the study setup did not utilize sufficient surface for monitoring the entire range of respiratory motion. For instance, both thoracic and abdominal motion were not fully monitored during the study. Since the study focused on the tumor position repeatability in breath‐hold, we utilized the ROI which was visible and reproducible during the study. Further investigations are needed regarding the monitoring location and size corresponding to the tumor position.

The applicability of the proposed strategy was presented by evaluating the residual motion of the tumors and the donut‐shaped imaging surrogates on intra‐fractional breath‐hold CBCTs. The small residual motion from the study demonstrates the clinical suitability of the proposed respiratory management strategy for Ethos CT‐STAR/SBRT.

## 5 CONCLUSION

We demonstrated the intra‐fractional motion‐managed treatment strategy in the Ethos CT‐STAR/SBRT using optical surface imaging. While the controlled residual tumor motion measured at 3.5 mm exceeded the predetermined setup value of 2 mm, it is important to note that this motion still fell within the clinically acceptable range defined by the PTV margin of 5 mm. Therefore, additional caution is needed with intra‐fractional motion management in Ethos CT‐STAR/SBRT using the optical surface imaging.

## AUTHOR CONTRIBUTIONS

Taeho Kim, Kendall Kiser, Eric Laugeman, Joshua Schiff, Shanti Marasini, Alex Price, Michael Gach, Nels Knutson, Casey Hatscher, and Geoffrey Hugo contributed to developing the systems, data collection, analysis, and writing of the manuscript. Pamela Samson, Clifford Robinson, and Lauren Henke contributed to data collection, and design of data analysis and provided clinical assistance. All authors discussed the results and contributed to the final manuscript.

## CONFLICT OF INTEREST STATEMENT

The authors declare no conflicts of interest.

## Data Availability

The data that support the findings of this study are available from the corresponding author upon reasonable request.
